# Triptolide attenuates irritable bowel syndrome via inhibiting ODC1

**DOI:** 10.1186/s12876-023-02847-8

**Published:** 2023-06-12

**Authors:** Ning Zhu, Liuyan Zhu, Xueliang Zhang, Chengbin Huang, Wenjun Xiang, Bingwu Huang

**Affiliations:** 1 Department of Cardiology, The Third Affiliated Hospital of Shanghai University (Wenzhou People’s Hospital), The Wenzhou Third Clinical Institute Affiliated to Wenzhou Medical University, No. 299 Guan Road, Wenzhou, 325000 Zhejiang Province People’s Republic of China; 2 Department of General Practice, The Third Affiliated Hospital of Shanghai University (Wenzhou People’s Hospital), The Wenzhou Third Clinical Institute Affiliated to Wenzhou Medical University, No. 299 Guan Road, Wenzhou, 325000 Zhejiang Province People’s Republic of China; 3 Department of Orthopedic Surgery, The Second Affiliated Hospital, Yuying Children’s Hospital of Wenzhou Medical University, 109 Xueyuan West Road, Wenzhou, 325000 Zhejiang Province People’s Republic of China; 4 Department of Pathology, The Third Affiliated Hospital of Shanghai University (Wenzhou People’s Hospital), The Wenzhou Third Clinical Institute Affiliated to Wenzhou Medical University, No. 299 Guan Road, Wenzhou, 325000 Zhejiang Province People’s Republic of China; 5 Department of Anesthesiology and Perioperative Medicine, The Second Affiliated Hospital, Yuying Children’s Hospital of Wenzhou Medical University, 109 Xueyuan West Road, Wenzhou, 325000 Zhejiang Province People’s Republic of China

**Keywords:** Triptolide, Irritable bowel syndrome, Inflammation, Ornithine decarboxylase 1

## Abstract

**Background:**

Irritable bowel syndrome (IBS) is a chronic disorder of the gut-brain axis with significant morbidity. Triptolide, an active compound extracted from Tripterygium wilfordii Hook F (TwHF), has been widely used as a major medicinal herb in the treatment of inflammatory disease.

**Methods:**

The chronic-acute combined stress (CAS) stimulation was used to establish IBS rat model. The model rats were then gavaged with triptolide. Forced swimming, marble-burying, fecal weight and abdominal withdrawal reflex (AWR) score were recorded. Pathologic changes in the ileal and colonic tissues were validated by hematoxylin and eosin staining. The inflammatory cytokines and Ornithine Decarboxylase-1 (ODC1) in the ileal and colonic tissues were performed by ELISA and WB.

**Results:**

Triptolide didn’t have antidepressant- and antianxiety- effects in rats caused by CAS, but decreased fecal weight and AWR score. In addition, Triptolide reduced the release of IL-1, IL-6, and TNF-α and the expression of ODC1 in the ileum and colon.

**Conclusion:**

The therapeutic efficacy of triptolide for IBS induced by CAS was revealed in this study, which may be related to the reduction of ODC1.

**Supplementary Information:**

The online version contains supplementary material available at 10.1186/s12876-023-02847-8.

## Introduction

Irritable bowel syndrome (IBS) is a chronic disorder of the gut-brain axis with a rising global incidence and results in significant morbidity. The disease contributes low work productivity, and high healthcare costs because of intestinal symptoms and its complications [1]. As is well known, IBS does not represent abnormol structural or biochemical basis. Hence, therapeutic strategies for IBS are often focused on the predominant, or most troublesome, symptom the patient experiences, rather than targeting underlying pathophysiology. Current therapeutic interventions for IBS are not sufficiently effective. The better understanding of the potential underlying mechanisms involved in the pathophysiology of IBS will bring new hope for future effective treatments. Recently, inflammation is considered to play a crucial role in development of IBS [2]. Particularly, studies have shown the persistence of mucosal inflammation and aberrant T cell activation trigger IBS [3]. And the research has reported that increasing the intake of anti-inflammatory dietary factors and reducing the intake of pro-inflammatory factors may be contributed to reducing the incidence of IBS [4]. Therefore, inhibition of inflammation is a promising strategy for IBS treatment.

Traditional Chinese medicine (TCM) has evolved over several thousands of years and has been proven to be effective in the treatment of digestive diseases. Triptolide is the main active compound purified from the Chinese herb Tripterygium wilfordii Hook.F and has multiple pharmacological properties such as immunomodulatory, anti-inflammatory, anti-proliferation, and antioxidant activities [5, 6]. The research has shown that triptolide could modulate the infiltration of macrophages and neutrophils and reduce the expression of proinflammatory factors [7]. Intraperitoneally administered triptolide reportedly ameliorates 2,4,6-trinitrobenzene sulfonic acid (TNBS)-induced colonic fibrosis of rats [8]. And the research found that triptolide could improve the colonic inflammatory response of interleukin-10 deficient mice [9]. Furthermore, triptolide and its derivative could protect mice from dextran sulfate sodium (DSS)-induced colitis [10, 11]. Based on these studies, we proposed that triptolide might play beneficial effects in the treatment of IBS through the therapeutic action against chronic inflammation.

Ornithine decarboxylase-1(ODC1) is the rate-limiting enzyme in the process of polyamine biosynthesis, which can catalyze the decarboxylation of ornithine to putrescine, regulate the level of polyamines and participate in the regulation of cellular life activities. ODC1 is closely related to inflammatory diseases [12, 13]. Recent studies have indicated that ODC1 can increase promote colitis and colitis related cancer by inhibiting stimulate epithelial repair, antimicrobial defense, and antitumoral immunity. However, ODC1 blockage can significantly reduce the production of inflammatory factors and improve the severity of colitis in mice [14, 15]. Intestinal inflammatory response is an important pathological change in IBS, and continuous inflammatory response can disrupt the intestinal mucosal barrier function, which is an important cause of diarrhea, visceral hypersensitivity, and pain in IBS patients [16, 17]. Therefore, ODC1 may modulate inflammation and subsequently IBS development.

In the present study, protective effects and underlying mechanism of triptolide on chronic acute combined stress (CAS)-induced IBS in rats was explored.

## Materials and methods

### Animal model and treatment

A total of 18 adult male Sprague-Dawley (SD) rats, weighing 200–220 g, were obtained from the Experimental Animal Center of the Zhejiang Province (Hangzhou, China). The rats were fed in groups of 3 per cage with a room temperature of 24 ± 1 °C and humidity of 50 ± 10%, and they were maintained on a 12-h light/dark cycle with water and food available ad libitum. After all rats were acclimatized to laboratory conditions for 1 week, the 6 rats per group in each were used to conduct behavior tests. All experiments were approved by Wenzhou Medical University Animal Care and Use Committee (Code of Ethics: wydw2022-0138) and were conducted by the National Institutes of Health Guide for Care and Use of Laboratory Animals. Triptolide was purchased from MedChemExpress, which was dissolved in DMSO and diluted with olive oil. All the rats were randomly divided into 3 groups (n = 6 each): the normal group administered via gavage of the same volume of olive oil, the IBS group and the IBS treated via gavage of triptolide (100 µg/kg/day) [18, 19]. 5 weeks later, behavioral tests and visceral sensitivity of the bowel were performed. Then the rats were anesthetized by i.p. injection of pentobarbital sodium, and their ileum and colon were harvested for further experiments. Finally, the rats were sacrificed by injecting excessive pentobarbital sodium.

### Chronic Acute combined stress (CAS) model

The rat in the CAS group and the treatment group was exposed to the following 7 stressors in random order with minor modifications: 4 °C cold environment for 5 min, overnight illumination for 12 h, water deprivation for 24 h, 40 °C hot environment for 10 min, tail clamp for 3 min, food deprivation for 24 h and bedding damp for 4 h. The stressors were presented randomly during 1 week and then repeated for 5 weeks [20]. On day 36, 3 h of acute restraint stress was given to each of these rats. Control rats were left undisturbed in the cages throughout the 5 weeks except for general handling.

### Forced swimming test

Rats were subjected a swimming-stress session for 15 min (pre-test), 24 h before being individually placed in glass cylinders (40 cm height, 18 cm diameter) filled with water (24 ± 1 °C; depth 23 cm) for 5 min (test). Each rat was supposed to be immobile when it ceased struggling and remained floating motionless in the water, making only small movements necessary to keep its head above water. And the immobility time of each rat was recorded during 5 min test period [21].

### Marble-burying test

Rats were placed individually in transparent propylene cages (40 × 24 × 20 cm) containing 5 cm deep sawdust and 9 clean glass marbles (diameter 23 mm) equally spaced along the wall. 10 min later, animals were removed, the number of marbles at least one-half buried in the sawdust was recorded [22].

### Intestinal tract motility

The weight of fecal pellets expelled during 1 h restraint period was used as an indirect measure of intestinal tract motility. Free access to water and food was given until the beginning of the procedure. This method was similar to that described previously, but with minor modifications [23].

### Abdominal Withdrawal Reflex (AWR) testing in rats

The visceral hypersensitivity is an essential characteristic feature of IBS. Visceral hypersensitivity responses to colorectal distension (CRD) were assessed by AWR scores, as described previously [24]. Rats in all the groups fasted on the day before the experiments, but the water was provided ad libitum. The balloon was constructed from a latex glove finger (6 cm of length) attached to a balloon dilator (2 mm of diameter), connected via a three-way pipe connector to a syringe pump and a sphygmomanometer. Rats were first anesthetized with isoflurane, and the balloon coating with vaseline oil was inserted into the distal colon with the distal tip 1 cm from the anal verge and secured by taping the attached tubing to the rat’s tail. The rats were then allowed to wake up and adapt for 30 min. Graded strengths of CRD at 20, 40, 60, and 80 mmHg were applied at 4 min intervals and kept inflation for 20 s at a time to produce different intensities of visceral pain. AWR responses were measured by blind observers who determined scores according to the following scales: 0, no behavioral response to CRD; 1, brief head movement followed by immobility; 2, contraction of abdominal muscles; 3, lifting of abdomen; 4, body arching and lifting of pelvic structures. The measurements were repeated 5 times for each intensity level and the data for each rat were averaged. AWR test was performed on day 37 after forced swimming test.

### Histological evaluation

Ileum and colon tissues obtained from rats were fixed with 4% paraformaldehyde, embedded in paraffin. These samples were sectioned at 4 μm and stained with haematoxylin-eosin (HE). Morphologyical analysis was conducted with light microscope (40X) (Nikon, Japan).

#### Determination of inflammatory markers IL-1, IL-6, and TNF-α

The IL-1, IL-6, and TNF-α Active ELISA (Active Motif, USA) kit was used to measure the binding activity of free IL-1, IL-6, and TNF-α in ileum and colon. The ileum and colon tissues adjacent to the cecum were collected and kept in -80 °C refrigerator, and IL-1, IL-6, and TNF-α activation assay were completed according to the kit instructions.

### Western blot analysis

The ileum and colon tissues were lysed with radio immunoprecipitation assay (RIPA) buffer containing protease and phosphatase inhibitors, and then centrifuged at 12,000 rpm for 30 min at 4 °C. And 30 µg protein samples were separated by electrophoresis on 10% SDS-PAGE gels. After electrophoresis, proteins from the gels were transferred onto polyvinylidene difluoride (PVDF) membranes and blocked with 5% dried milk in Tris-buffered saline (TBS) containing 0.1% Tween 20 for 2 h at room temperature. The membranes were incubated with the appropriate primary antibodies, i.e., Ornithine decarboxylase-1 (ODC1) (1:1000; Proteintech Group, USA), GAPDH (1:1000; Cell signaling technology, USA), overnight at 4 °C. After washing, the PVDF membranes were incubated with the horseradish peroxidase-linked secondary antibodies (goat anti-rabbit IgG-HRP, 1:2000; Cell signaling technology, USA) for 2 h at room temperature. Labeled protein bands were visualized by using ECL kit (Advansta, USA) and quantified using Image J software (National Institutes of Health, USA).

### Statistical analysis

All data were expressed as mean ± standard deviation (SD). The data were analyzed statistically using independent-samples t-test and one-way analysis of variance (ANOVA), followed by a post hoc Tukey’s test. P < 0.05 was considered statistically significant.

## Results

### The established IBS model in rats and triptolide had no effects on behaviors

**Fig. 1:**
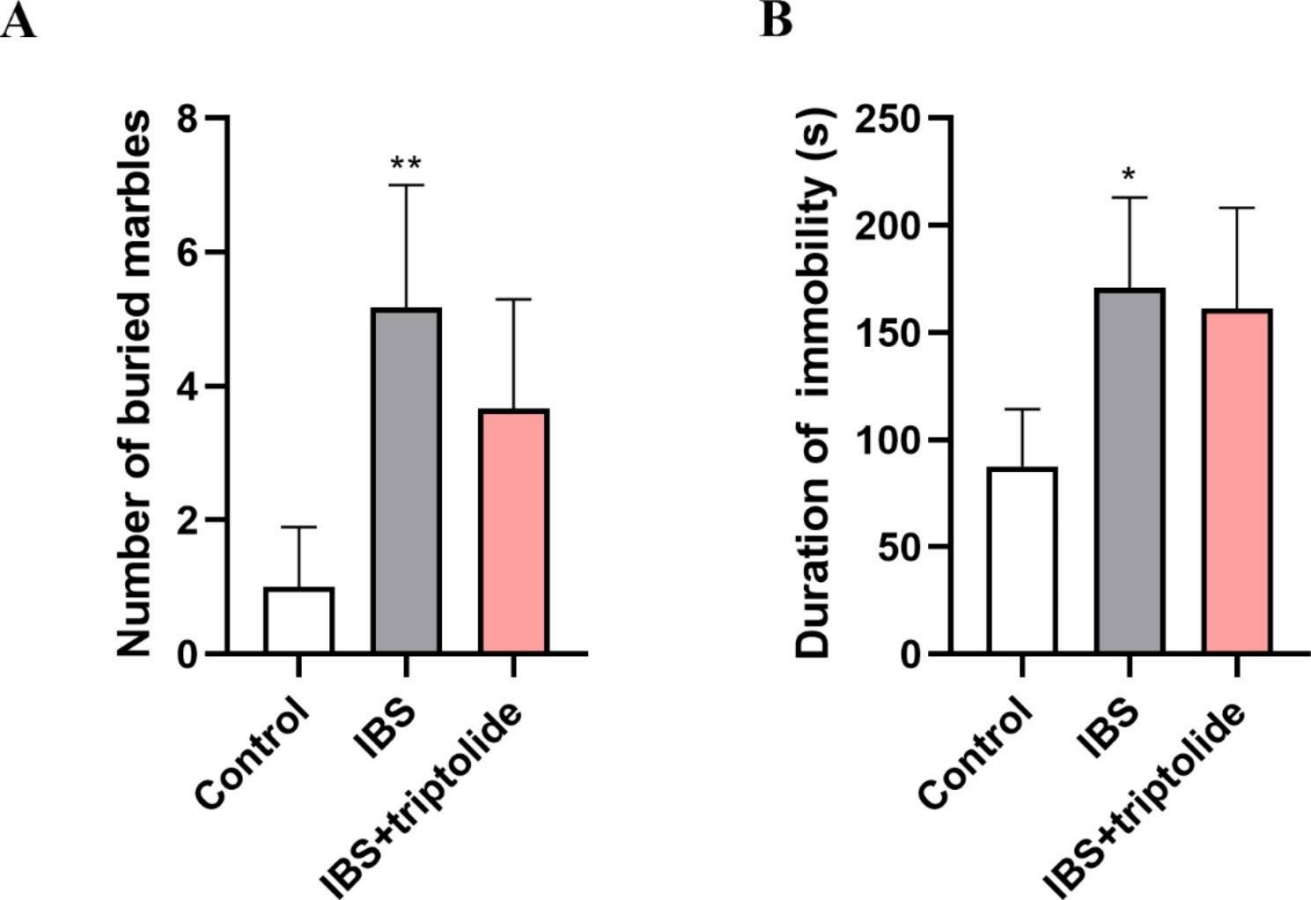
The effects of triptolide administration on behavioral symptoms in rats with CAS-inudced IBS. The the marbleburying tests (A) and immobility time in the forced swimming (B) were performed to investigate anxiety- and depressant- like behaviors. The rats were either subjected to chronic acute combined stress for 35 days or left undisturbed (control group). Results are expressed as mean ± SD (n = 6). *P < 0.05 and **P < 0.01 vs. Control group

The potential anti-anxiety- and antidepressant- like effects of triptolide were evaluated in marble-burying test and the forced swimming tests. Forced swimming test showed that CAS induced IBS group had longer immobility time than that of control group (P < 0.05). Rats treated with triptolide with the increase of the immobility time were similar to those of CAS induced IBS group (P<0.05). Marble-burying test showed the similar results (control group vs. IBS group, P < 0.05; IBS group vs. IBS + triptolide group, P<0.05) (Fig. 1).

### Triptolide decreased visceral hypersensitivity of bowel

**Fig. 2:**
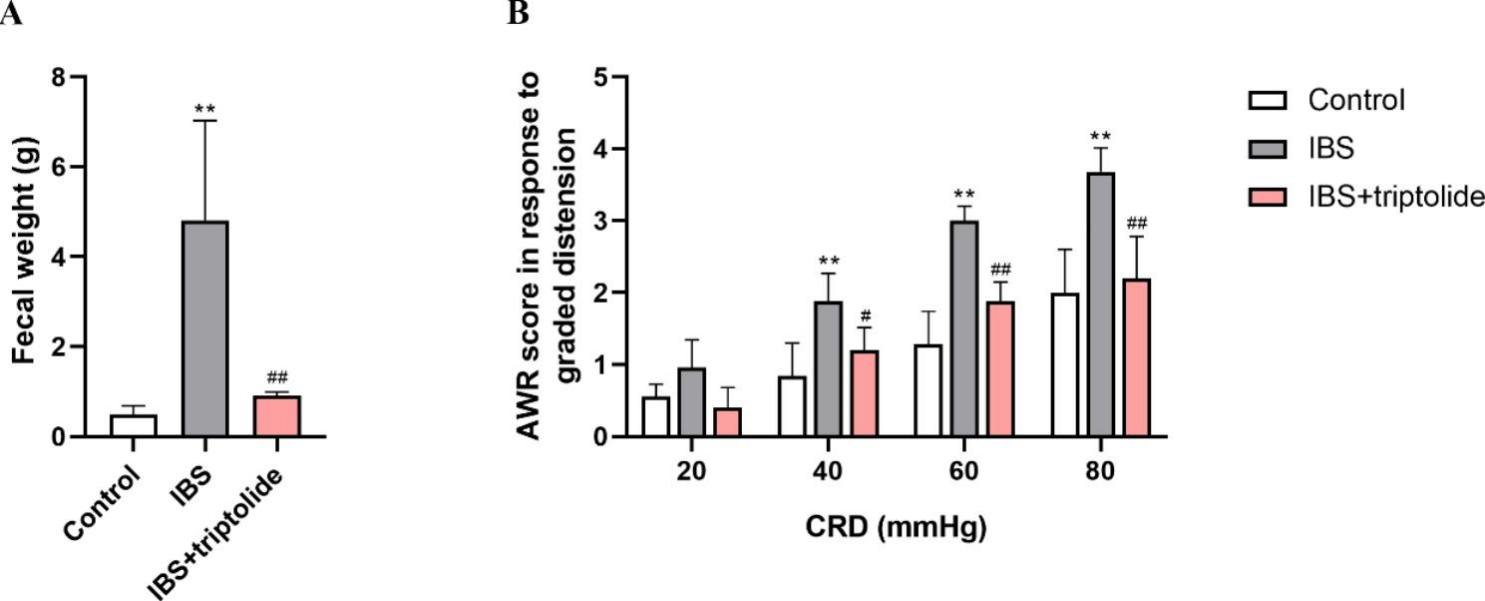
The effects of triptolide administration on visceral hypersensitivity of bowel in rats with CAS-inudced IBS. The fecal weight (A), n = 4 and AWR score in the CRD testing (B), n = 6 was carried out to evaluate visceral hypersensitivity of bowel. The rats were either subjected to chronic acute combined stress for 35 days or left undisturbed (Control group). Results are expressed as mean ± SD. **P < 0.01 vs. Control group, ^#^*P* < 0.05 and ^##^*P* < 0.01 vs. IBS group. CRD, colorectal distension; AWR, abdominal withdrawal reflex

**Fig. 3:**
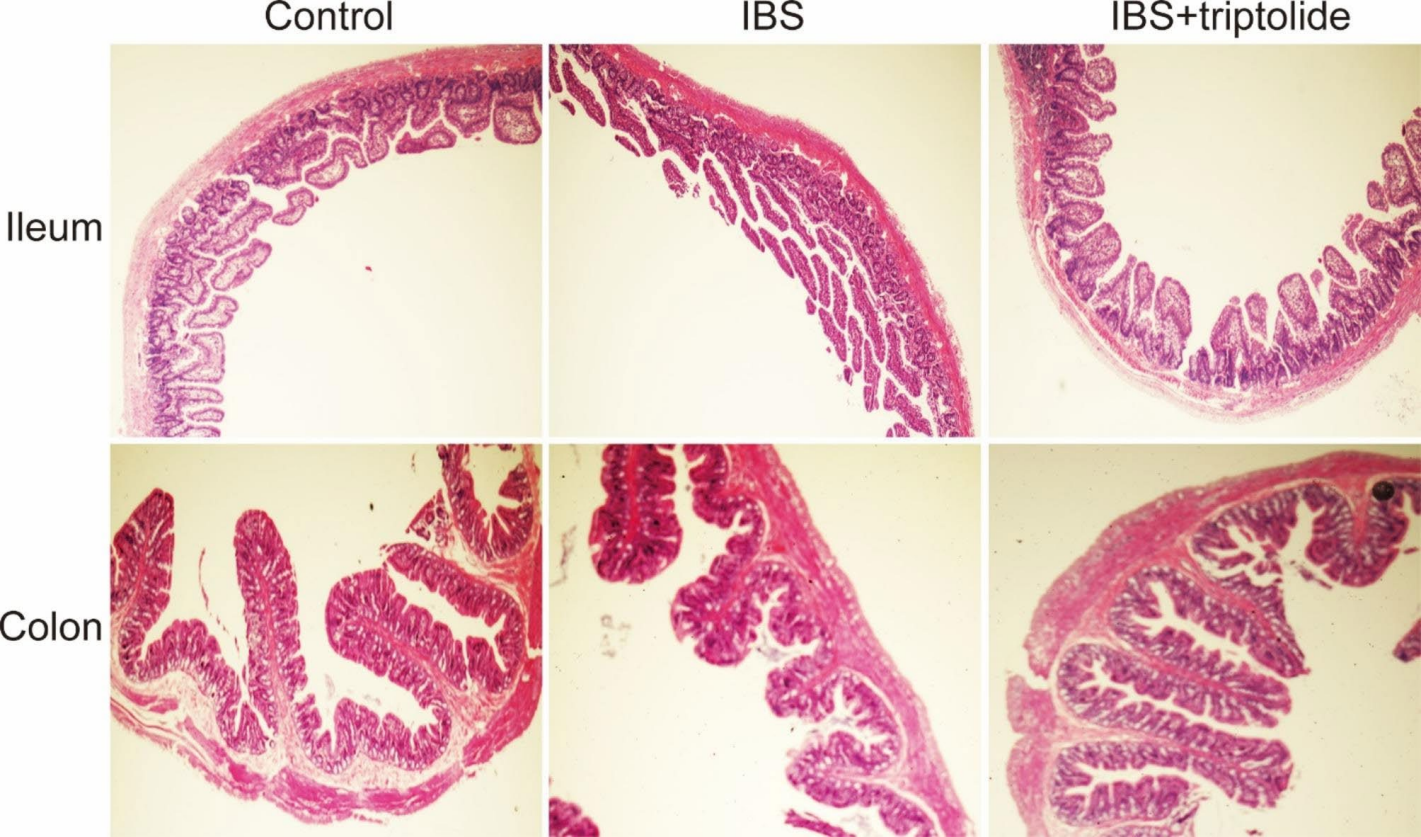
The intestinal structural changes after treatment with triptolide in rats with CAS-inudced IBS. HE was used to stain the ileum and colon of rats in each group (40X). HE, hematoxylin and eosin

Fecal weight was significantly higher in IBS group compared with control group (P < 0.01), which was reversed by triptolide (P < 0.01). AWR scores were strikingly increased from low pressure (40mmHg) to high pressure (80mmHg) (P < 0.01) in IBS group, while triptolide remarkably reduced the increase (P < 0.01) (Fig. 2). There was no significant change in histological analysis among the control group, IBS group and IBS + triptolide group (Fig. 3). The results of forced swimming test, marble-burying test and AWR scores, as well as histological analysis suggested that CAS successfully induced IBS model in rats.

### Triptolide exerted anti-inflammatory effects in the ileum and colon

**Fig. 4:**
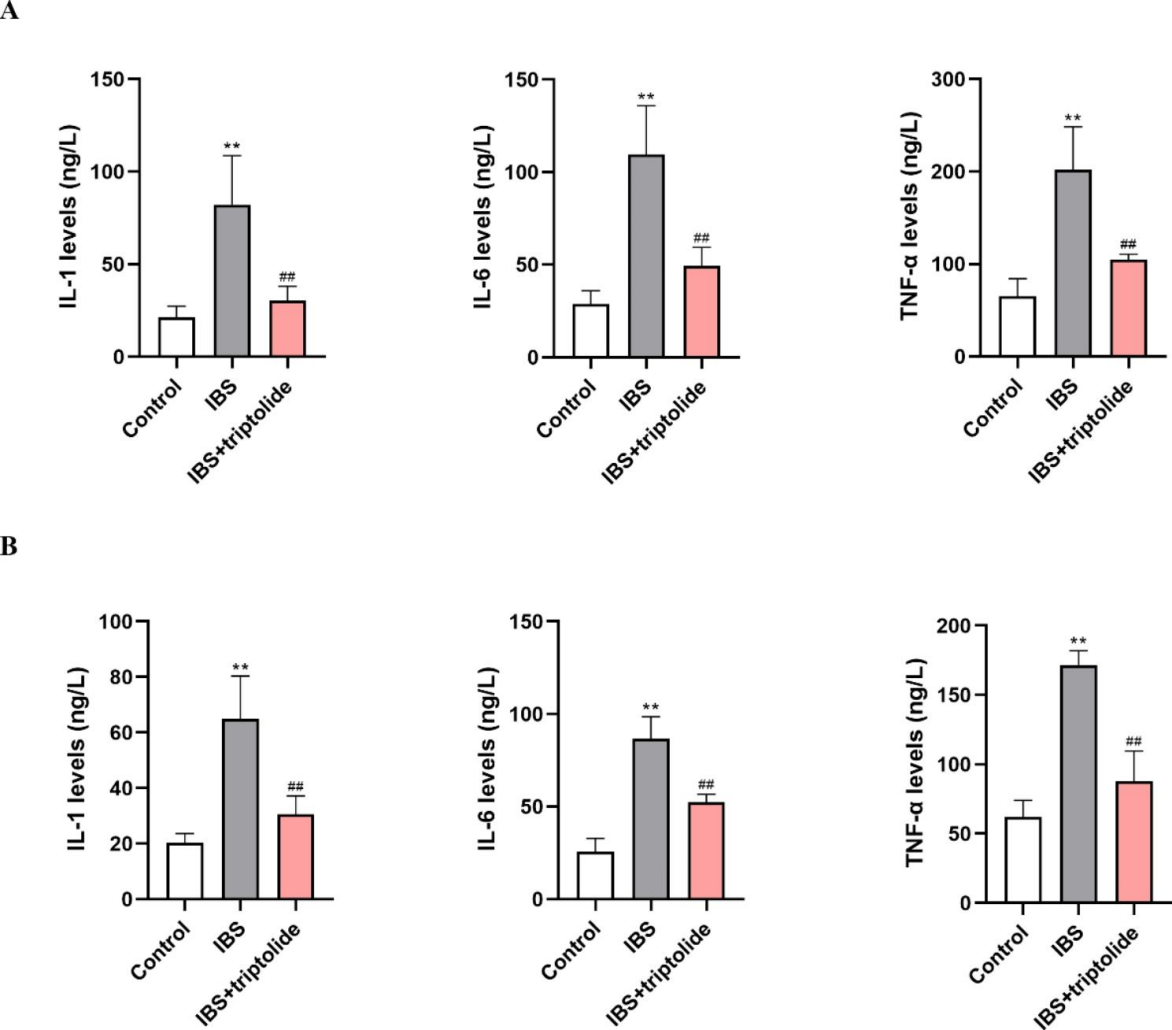
The effects of triptolide administration on inflammatory cytokines expression in rats with CAS-inudced IBS. ELISA was conducted to measure the expression of IL-1, IL-6, and TNF-α in the ileum (A) and colon (B) in each group. Results are expressed as mean ± SD (n = 6). **P < 0.01 vs. Control group, ^##^*P* < 0.01 vs. IBS group

Compared with control group, IBS group has high levels of IL-1, IL-6, and TNF-α (P < 0.01). However, triptolide reduced the release of IL-1, IL-6, and TNF-α compared with IBS group (P < 0.01) (Fig. 4).

### Triptolide reversed ODC1 protein level in the ileum and colon

**Fig. 5:**
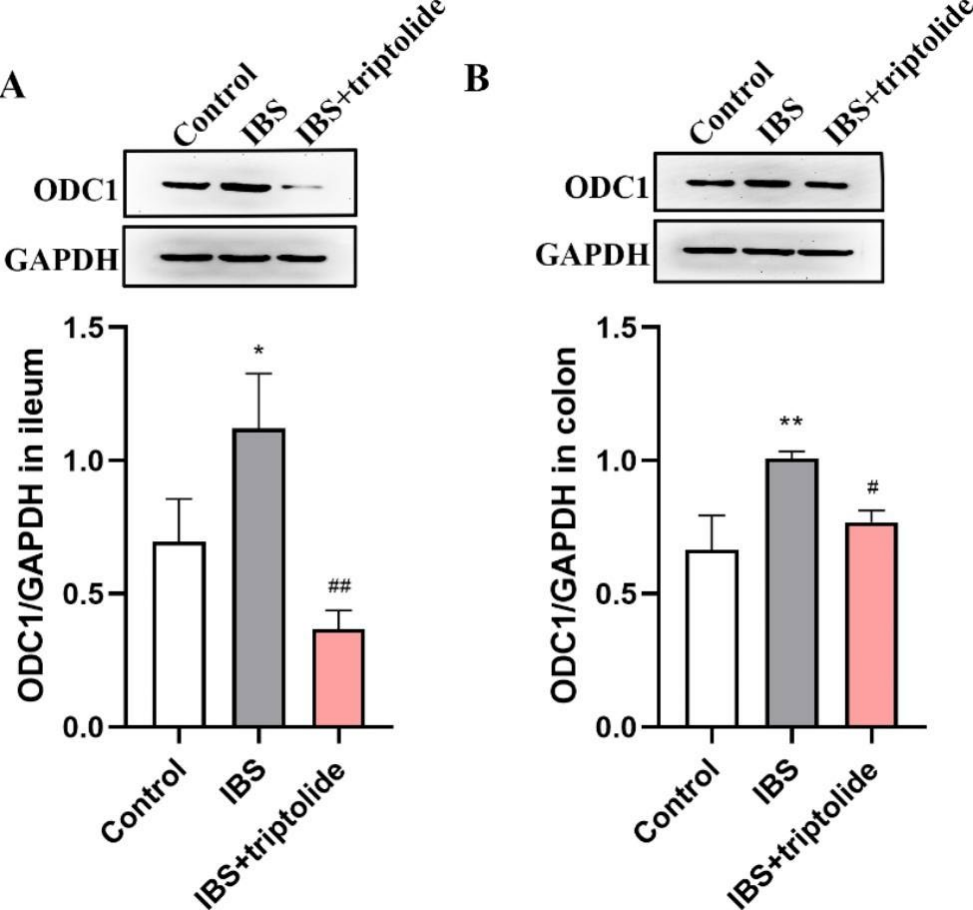
The effects of triptolide administration on ODC expression in rats with CAS-inudced IBS. Western blot analysis was carried out to detect the protein expression of ODC in the ileum (A) and colon (B) normalized to GAPDH. The samples derive from the same experiment and that blots were processed in parallel. Results are expressed as mean ± SD (n = 3). *P < 0.05, **P < 0.01 vs. Control group, and ^#^*P* < 0.05, ^##^*P* < 0.01 vs. IBS group

ODC1 was increased in the ileum (P < 0.05) and colon induced by CAS (P < 0.01), while triptolide treatment inhibited the increase (P < 0.05) (Fig. 5).

## Discussion

This study indicated that though triptolide didn’t attenuated the behavioral symptoms, but still ameliorate visceral hypersensitivity in chronic-acute combined stress (CAS)-induced IBS in rats. In addition, triptolide decrease inflammatory cytokines and ODC1 expression. This study suggested triptolide is a promising drug for IBS treatment and ODC1 may be an effective target.

As one of the most important functional bowel disorders, IBS is responsible for more than 10% of bowel symptoms in the global adult population based on population-based surveys [25]. IBS treatment often focuses on the predominant symptoms, including fiber supplements, probiotics, antidepressants, and 5-hydroxytryptophan 3 receptor antagonists [26]. However, these treatments don’t sufficiently affect the natural history of IBS in the long term [27]. TCM has become an important choice for pharmaceutical research and drug discovery. Recently, a few TCMs were used to treat IBS, such as Shenling Baizhu and Fuzi-Lizhong [28, 29].

Since IBS is considered as a brain–gut disorder, gut–brain dysfunction plays a crucial role in the progression of IBS. Chronic-acute induced stress in animal models were previously utilized to determine the molecular mechanisms underlying not only emotional disorders, but also many other extracerebral disease such as atherosclerosis, hepatic injury, blood pressure variability, etc [30–32]. These models also promoted drug discovery. Animals subjected to CAS result in psychiatric disorders and visceral hypersensitivity, especially in the ileum and colon [13, 20], which was considered as IBS model. It is well known that visceral sensitivity is responsible for the pathophysiology of functional bowel disease. Furthermore, hypersensitivity contributes to abdominal pain associated with defecation or a change in the gastrointestinal tract [33]. In the present study, we applied the CAS procedure to drive the symptoms of IBS and bowel disorders. In line with the clinical pathology of IBS [34, 35], the pathological manifestations were not observed in the ileum and colon.

Though the relation between inflammation and IBS has not been well identified, post-infectious changes, chronic infections, and immune activation were traditionally described as the underlying mechanisms triggering IBS [36]. Low-grade inflammation (LGI) is defined as inflammatory changes with a lower pathological degree in the intestine, the degree of which is higher than the “physiological inflammation” of the normal intestinal mucosa but without significant external manifestation of inflammation. LGI is an important pathological change of IBS. Continuous LGI causes the damage to the intestinal mucosal barrier function, which results in diarrhea, visceral hypersensitivity, and pain in IBS [16, 17]. 2021 the American College of Gastroenterology Clinical Guideline also recommended two fecal-derived markers of intestinal inflammation, fecal lactoferrin and fecal calprotectin, as well as ESR and CRP, were diagnostically useful for IBS [37]. The most studies regarding IBS focused on gut bacterial­mediated inflammation. The Previous studies showed that water avoidance stress also could cause visceral hypersensitivity and release of inflammatory factors, such as IL-1β, and IL-18 in the colon [38]. The infiltration of inflammatory cells was not found in the ileum and colon. Noticeably, our data showed inflammatory cytokines including IL-1. IL-6 and TNFα were remarkably increased in ACS-induced IBS. The data suggested that CAS-induced low-grade inflammation.

Triptolide, a small molecule purified from the TwHF, also was considered as a potent anti-inflammatory agent [39]. Triptolide was proven to have therapeutic effects on rheumatoid arthritis [40]. In addition, it was shown triptolide ameliorates colitis and inflammatory responses [11]. Triptolide also was reported to improve cognitive dysfunction with vascular dementia [41]. More importantly, triptolide was identified as a potent anti-depressive drug [18]. Tring to killing two birds with one stone, the stone is the disease (‘’IBS’’), and birds are pathological mechanisms (‘’brain-gut axis and inflammation’’). Indeed, triptolide treatment attenuated IBS-induced visceral hypersensitivity and inflammatory cytokines in the ileum and colon. However, triptolide treatment didn’t reduce the behavioral symptoms of IBS induced by CAS. The result indicated brain-gut axis isn’t involved in the effects of triptolide on IBS. And the sole inhibition of targeting inflammation can be recognized as an effective strategy.

ODC1 is the first and rate-limiting enzyme in the biosynthesis of polyamines and implicated in the generation of polyamines by the decarboxylation of ornithine [42]. Polyamines regulate various physiological processes such as cell proliferation and apoptosis, DNA stabilization, transcription and translation, etc [43, 44]. The overexpression of ODC1 in multiple cancerous tissues contributes to tumor growth via generation of increased polyamines [45–47]. Recently, the role of ODC1 in modulating gastric and colonic inflammation in macrophages after bacterial infections were reported [48]. Moreover, it was found that macrophage ODC1 promotes colitis and colitis-related colon carcinogenesis [15]. Thus, we propose ODC1 may play a key role in IBS and ODC1 is involved in the effects of triptolide. In the present study, the increase of ODC1 in the ileum and colon induced by CAS was reduced by triptolide treatment.

Our study still had the following limitations. Firstly, our study clarified the effect of triptolide on CAS-induced IBS in rats, other IBS models, such as colon administration and maternal isolation should be performed to confirmed these results. Secondly, the role of ODC1 in IBS should be further confirmed by ODC1 knockout mice. Finally, these results are based on animal experiments, and the role of ODC1 in the treatment of IBS in the clinical setting remain be validated in future.

## Conclusion

In summary, our data demonstrated that triptolide could attenuate CAS-induced IBS by inhibiting inflammatory cytokines but not via the brain-gut axis, and the underlying mechanism was correlated to the decrease of ODC1.

## Electronic supplementary material

Below is the link to the electronic supplementary material.

Supplementary Material 1

## Data Availability

The data used to support the findings of this study are included within the article.

## References

[1] Colomier E, Algera J, Melchior C (2020). Pharmacological therapies and their clinical targets in irritable bowel syndrome with Diarrhea. Front Pharmacol.

[2] Sinagra E, Morreale GC, Mohammadian G, Fusco G, Guarnotta V, Tomasello G, Cappello F, Rossi F, Amvrosiadis G, Raimondo D (2017). New therapeutic perspectives in irritable bowel syndrome: Targeting low-grade inflammation, immuno-neuroendocrine axis, motility, secretion and beyond. World J Gastroenterol.

[3] Ng QX, Soh AYS, Loke W, Lim DY, Yeo WS (2018). The role of inflammation in irritable bowel syndrome (IBS). J Inflamm Res.

[4] Eslampour E, Ghanadi K, Aghamohammadi V, Kazemi AM, Mohammadi R, Vahid F, Abbasnezhad A (2021). Association between dietary inflammatory index (DII) and risk of irritable bowel syndrome: a case-control study. Nutr J.

[5] Wang Y, Wang B, Yang X (2020). The study of Cellular mechanism of Triptolide in the treatment of Cancer, Bone loss and Cardiovascular Disease and Triptolide’s toxicity. Curr Stem Cell Res Therapy.

[6] Jiang X, Cao G, Gao G, Wang W, Zhao J, Gao C (2021). Triptolide decreases tumor-associated macrophages infiltration and M2 polarization to remodel colon cancer immune microenvironment via inhibiting tumor-derived CXCL12. J Cell Physiol.

[7] Yang J, Tang X, Ke X, Dai Y, Shi J. Triptolide suppresses NF-κB-Mediated inflammatory responses and activates expression of Nrf2-Mediated antioxidant genes to Alleviate Caerulein-Induced Acute Pancreatitis. Int J Mol Sci 2022, 23(3). 10.3390/ijms23031252 PMC8835869 35163177

[8] Tao Q, Wang B, Zheng Y, Li G, Ren J (2015). Triptolide ameliorates colonic fibrosis in an experimental rat model. Mol Med Rep.

[9] Wei X, Gong J, Zhu J, Niu L, Zhu W, Li N, Li J (2008). Therapeutic effects of triptolide on interleukin-10 gene-deficient mice with colitis. Int Immunopharmacol.

[10] Fu J, Zang Y, Zhou Y, Chen C, Shao S, Shi G, Wu L, Zhu G, Sun T, Zhang D (2021). Exploring a novel triptolide derivative possess anti-colitis effect via regulating T cell differentiation. Int Immunopharmacol.

[11] Tang B, Zhu J, Zhang B, Wu F, Wang Y, Weng Q, Fang S, Zheng L, Yang Y, Qiu R (2020). Therapeutic potential of Triptolide as an anti-inflammatory Agent in Dextran Sulfate Sodium-Induced Murine Experimental Colitis. Front Immunol.

[12] Downs IA, Aroniadis OC, Kelly L, Brandt LJ (2017). Postinfection Irritable Bowel Syndrome: the links between gastroenteritis, inflammation, the Microbiome, and Functional Disease. J Clin Gastroenterol.

[13] Xu Y, Cui SY, Ma Q, Shi J, Yu Y, Li JX, Zheng L, Zhang Y, Si JM, Yu YC (2018). Trans-resveratrol ameliorates Stress-Induced Irritable Bowel Syndrome-Like Behaviors by Regulation of Brain-Gut Axis. Front Pharmacol.

[14] Peng V, Cao S, Trsan T, Bando JK, Avila-Pacheco J, Cleveland JL, Clish C, Xavier RJ, Colonna M (2022). Ornithine decarboxylase supports ILC3 responses in infectious and autoimmune colitis through positive regulation of IL-22 transcription. Proc Natl Acad Sci USA.

[15] Singh K, Coburn LA, Asim M, Barry DP, Allaman MM, Shi C, Washington MK, Luis PB, Schneider C, Delgado AG (2018). Ornithine Decarboxylase in Macrophages exacerbates colitis and promotes Colitis-Associated Colon carcinogenesis by impairing M1 Immune responses. Cancer Res.

[16] Kerckhove N, Scanzi J, Pereira B, Ardid D, Dapoigny M (2017). Assessment of the effectiveness and safety of ethosuximide in the treatment of abdominal pain related to irritable bowel syndrome - IBSET: protocol of a randomised, parallel, controlled, double-blind and multicentre trial. BMJ open.

[17] Beatty JK, Bhargava A, Buret AG (2014). Post-infectious irritable bowel syndrome: mechanistic insights into chronic disturbances following enteric infection. World J Gastroenterol.

[18] Hu X, Dong Y, Jin X, Zhang C, Zhang T, Zhao J, Shi J, Li J (2017). The novel and potent anti-depressive action of triptolide and its influences on hippocampal neuroinflammation in a rat model of depression comorbidity of chronic pain. Brain Behav Immun.

[19] Wang W, Mei XP, Chen L, Tang J, Li JL, Wu SX, Xu LX, Li YQ (2012). Triptolide prevents and attenuates neuropathic pain via inhibiting central immune response. Pain Physician.

[20] Zou N, Lv H, Li J, Yang N, Xue H, Zhu J, Qian J (2008). Changes in brain G proteins and colonic sympathetic neural signaling in chronic-acute combined stress rat model of irritable bowel syndrome (IBS). Translational research: the journal of laboratory and clinical medicine.

[21] Yu Y, Wu S, Li J, Wang R, Xie X, Yu X, Pan J, Xu Y, Zheng L (2015). The effect of curcumin on the brain-gut axis in rat model of irritable bowel syndrome: involvement of 5-HT-dependent signaling. Metab Brain Dis.

[22] Schneider T, Popik P (2007). Attenuation of estrous cycle-dependent marble burying in female rats by acute treatment with progesterone and antidepressants. Psychoneuroendocrinology.

[23] Barone FC, Deegan JF, Price WJ, Fowler PJ, Fondacaro JD, Ormsbee HS (1990). 3rd: cold-restraint stress increases rat fecal pellet output and colonic transit. Am J Physiol.

[24] Yang JM, Xian YF, Ip PS, Wu JC, Lao L, Fong HH, Sung JJ, Berman B, Yeung JH, Che CT (2012). Schisandra chinensis reverses visceral hypersensitivity in a neonatal-maternal separated rat model. Phytomedicine: Int J phytotherapy phytopharmacology.

[25] Lovell RM, Ford AC (2012). Global prevalence of and risk factors for irritable bowel syndrome: a meta-analysis. Clin Gastroenterol hepatology: official Clin Pract J Am Gastroenterological Association.

[26] Rivkin A, Rybalov S (2016). Update on the management of Diarrhea-Predominant Irritable Bowel Syndrome: Focus on Rifaximin and Eluxadoline. Pharmacotherapy.

[27] Ford AC, Forman D, Bailey AG, Axon AT, Moayyedi P (2008). Irritable bowel syndrome: a 10-yr natural history of symptoms and factors that influence consultation behavior. Am J Gastroenterol.

[28] Meng M, Bai C, Wan B, Zhao L, Li Z, Li D, Zhang S (2021). A Network Pharmacology-Based study on irritable Bowel Syndrome Prevention and Treatment utilizing Shenling Baizhu Powder. Biomed Res Int.

[29] Zhen Z, Xia L, You H, Jingwei Z, Shasha Y, Xinyi W, Wenjing L, Xin Z, Chaomei F (2021). An Integrated Gut Microbiota and Network Pharmacology Study on Fuzi-Lizhong pill for treating diarrhea-predominant irritable bowel syndrome. Front Pharmacol.

[30] Hinterdobler J, Schunkert H, Kessler T, Sager HB. Impact of Acute and chronic psychosocial stress on vascular inflammation. Antioxidants & redox signaling; 2021. 10.1089/ars.2021.0153 PMC8713271 34293932

[31] Heidari R, Niknahad H, Sadeghi A, Mohammadi H, Ghanbarinejad V, Ommati MM, Hosseini A, Azarpira N, Khodaei F, Farshad O (2018). Betaine treatment protects liver through regulating mitochondrial function and counteracting oxidative stress in acute and chronic animal models of hepatic injury. Biomed pharmacotherapy = Biomedecine pharmacotherapie.

[32] Farah VM, Joaquim LF, Bernatova I, Morris M (2004). Acute and chronic stress influence blood pressure variability in mice. Physiol Behav.

[33] Fei L, Wang Y (2020). microRNA-495 reduces visceral sensitivity in mice with diarrhea-predominant irritable bowel syndrome through suppression of the PI3K/AKT signaling pathway via PKIB. IUBMB Life.

[34] Collins SM (2016). The intestinal microbiota in the irritable bowel syndrome. Int Rev Neurobiol.

[35] Lacy BE, Patel NK. Rome Criteria and a Diagnostic Approach to Irritable Bowel Syndrome. J Clin Med 2017, 6(11). 10.3390/jcm6110099 PMC5704116 29072609

[36] Holtmann GJ, Ford AC, Talley NJ (2016). Pathophysiology of irritable bowel syndrome. lancet Gastroenterol Hepatol.

[37] Lacy BE, Pimentel M, Brenner DM, Chey WD, Keefer LA, Long MD, Moshiree B (2021). ACG Clinical Guideline: management of irritable bowel syndrome. Am J Gastroenterol.

[38] Yu LM, Zhao KJ, Wang SS, Wang X, Lu B (2019). Corticotropin-releasing factor induces inflammatory cytokines via the NLRP6-inflammatory cytokine axis in a murine model of irritable bowel syndrome. J Dig Dis.

[39] Tasneem S, Liu B, Li B, Choudhary MI, Wang W (2019). Molecular pharmacology of inflammation: Medicinal plants as anti-inflammatory agents. Pharmacol Res.

[40] Song X, Zhang Y, Dai E, Wang L, Du H (2020). Prediction of triptolide targets in rheumatoid arthritis using network pharmacology and molecular docking. Int Immunopharmacol.

[41] Yao P, Li Y, Yang Y, Yu S, Chen Y (2019). Triptolide improves cognitive dysfunction in rats with vascular dementia by activating the SIRT1/PGC-1α signaling pathway. Neurochem Res.

[42] Liu YC, Liu YL, Hsieh JY, Wang CH, Lin CL, Liu GY, Hung HC. Baicalein, 7,8-Dihydroxyflavone and myricetin as potent inhibitors of human ornithine decarboxylase. Nutrients 2020, 12(12). 10.3390/nu12123867 PMC7765794 33348871

[43] Somani RR, Rai PR, Kandpile PS (2018). Ornithine Decarboxylase Inhibition: a strategy to Combat various Diseases. Mini Rev Med Chem.

[44] Yurdagul A, Subramanian M, Wang X, Crown SB, Ilkayeva OR, Darville L, Kolluru GK, Rymond CC, Gerlach BD, Zheng Z (2020). Macrophage metabolism of apoptotic cell-derived arginine promotes continual efferocytosis and resolution of Injury. Cell Metabol.

[45] Kim HI, Schultz CR, Buras AL, Friedman E, Fedorko A, Seamon L, Chandramouli GVR, Maxwell GL, Bachmann AS, Risinger JI (2017). Ornithine decarboxylase as a therapeutic target for endometrial cancer. PLoS ONE.

[46] Gerner EW, Meyskens FL (2004). Polyamines and cancer: old molecules, new understanding. Nat Rev Cancer.

[47] Casero RA, Marton LJ (2007). Targeting polyamine metabolism and function in cancer and other hyperproliferative diseases. Nat Rev Drug Discovery.

[48] Hardbower DM, Asim M, Luis PB, Singh K, Barry DP, Yang C, Steeves MA, Cleveland JL, Schneider C, Piazuelo MB (2017). Ornithine decarboxylase regulates M1 macrophage activation and mucosal inflammation via histone modifications. Proc Natl Acad Sci USA.

