# Supplementary figures and images for: Triptolide attenuates irritable bowel syndrome via inhibiting ODC1
Source: BMC Gastroenterol. 2023 Jun 12;23:202. doi: 10.1186/s12876-023-02847-8 (PMC10258977; doi:10.1186/s12876-023-02847-8)

ODC1 in ileum


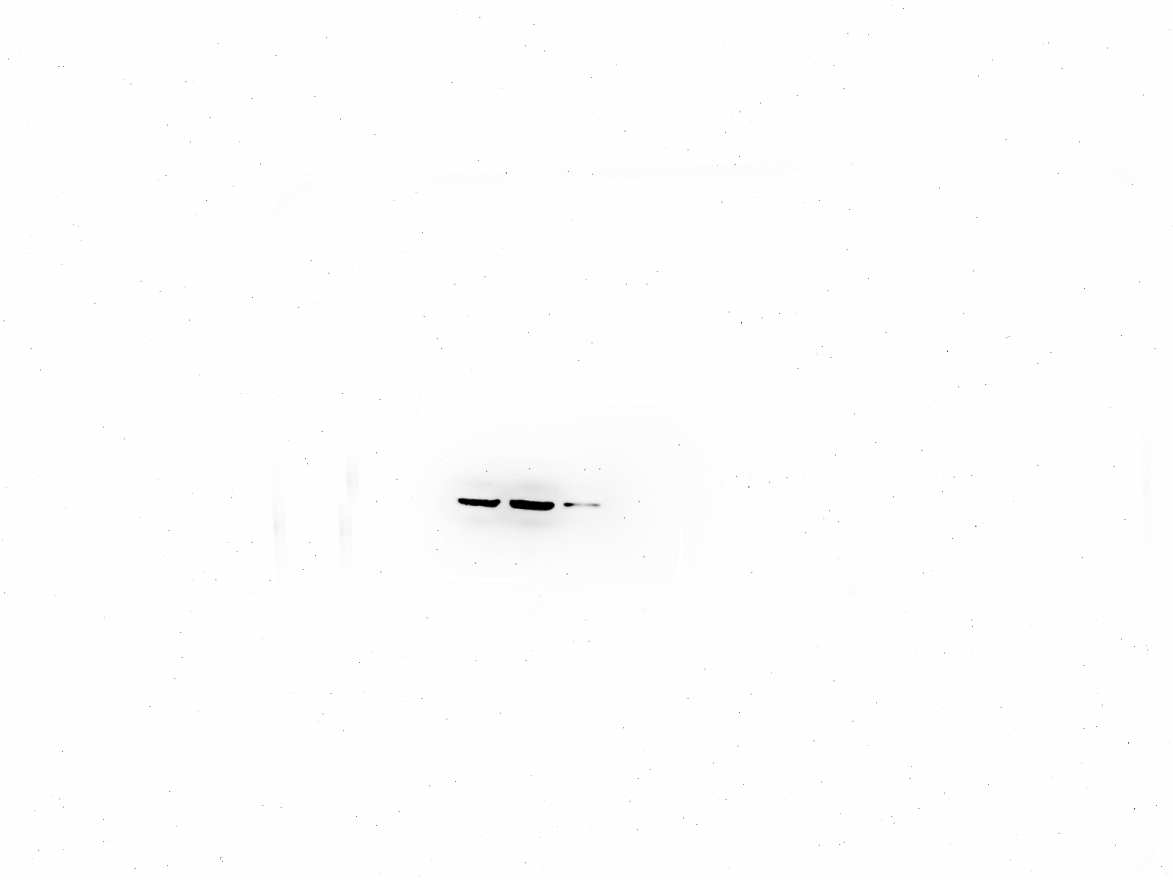





GAPDH in ileum







ODC1 in colon







GAPDH in colon

Supplement: Supplementary file 1 — Supplementary Material 1 [file 12876_2023_2847_MOESM1_ESM.docx]
